# Establishment of U-87MG Cellular Fibrosis as a Novel in Vitro Model to Analyze Glioblastoma Cells’ Sensitivity to Temozolomide

**DOI:** 10.3390/ijms26136121

**Published:** 2025-06-25

**Authors:** Valentina Lopardo, Roberta Maria Esposito, Antonio C. Pagano Zottola, Federica Santoro, Nicola Grasso, Alfonso Carotenuto, Annibale Alessandro Puca, Elena Ciaglia

**Affiliations:** 1 Department of Medicine, Surgery and Dentistry “Scuola Medica Salernitana”, University of Salerno, Via Salvatore Allende, 84081 Baronissi, Italy; vlopardo@unisa.it (V.L.); robeesposito@unisa.it (R.M.E.); apuca@unisa.it (A.A.P.); 2 Bordeaux Institute of Oncology, BRIC, INSERM, Unit U1312, University of Bordeaux, 33600 Pessac, France; antonio.pagano-zottola@u-bordeaux.fr; 3 Department of Pharmacy, University of Naples Federico II, 80131 Naples, Italy; federica.santoro@unina.it (F.S.); nicola.grasso@unina.it (N.G.); alfonso.carotenuto@unina.it (A.C.); 4 Cardiovascular Research Unit, IRCCS MultiMedica, 20138 Milan, Italy

**Keywords:** GBM, fibrosis, tumor-associated fibrotic alterations, chemoresistance

## Abstract

Glioblastoma (GBM), a highly malignant brain tumor, arises within a complex microenvironment that plays a critical role in facilitating tumor progression, ensuring survival, and enabling immune evasion, ultimately contributing to therapeutic resistance. Cancer-associated fibrosis is increasingly recognized as a key factor in the tumor pathophysiology, particularly in extracranial cancers, and reported therapeutic strategies in several cancers consist of the current use of the standard-of-care treatment combined with anti-fibrotic drugs. However, it remains unclear how the fibrotic changes associated with the GBM microenvironment contribute to the transformation of GBM from a chemosensitive state to a chemoresistant one. Here, we developed an in vitro model that mimics a fibrosis-like mechanism using the U-87MG GBM cell line. To achieve this, we identified the optimal experimental conditions (i.e., U-87MG cultured in serum-deprivation medium in the presence of recombinant TGF-B1 at 5 ng/mL for 72 h) that effectively induced fibrosis, as suggested by the counter-regulated expression of E- and N-cadherin and sustained levels of α-SMA and collagen I. As expected, U-87MG fibrotic cells were demonstrated to be more resistant to TMZ (predicted EC_50_ = 35 µM) as compared to the non-fibrotic counterpart (EC_50_ not achieved here; predicted EC_50_ = 351 µM). Accordingly, the anti-fibrotic uPAcyclin—a new derivative cyclic compound inspired as a A6 decapeptide drug—showed a significant cytotoxic effect, sensitizing resistant U-87MG fibrotic cells to TMZ. This highlights that targeting fibrosis may help to overcome TMZ resistance in GBM.

## 1. Introduction

Glioblastomas (GBMs) are the most common and deadly tumors among central nervous system (CNS)-associated malignancies. Unfortunately, for patients diagnosed with GBM, the median overall survival is only about 15 months.

In 2005, Stupp et al. introduced an approved standard treatment for GBM patients, widely known as “Stupp’s regimen” [1]. According to the original study, the Stupp protocol combines radiation therapy concomitant with temozolomide (TMZ) after surgical resection. This approach resulted in a significant survival improvement at 2 years (i.e., 26.5% 2-year survival with the Stupp protocol, compared to 10.4% 2-year survival with radiation therapy alone) [1].

TMZ, an imidazotetrazine derivative of the alkylating agent dacarbazine, functions as a prodrug for the anti-cancer drug Temodar [2]. At the molecular level, TMZ induces cell cycle arrest at the G2/M phase, ultimately triggering apoptosis [3]. The cytotoxic effect of TMZ is mediated by its addition of methyl groups at N7 and O6 on guanines and at O3 on adenines in genomic DNA, leading to the insertion of aberrant nucleotide bases during subsequent DNA replication and resulting in cell death [3]. Furthermore, radiation therapy (RT) primarily causes DNA double-strand breaks in GBM cells. After 20 years, the Stupp regimen remains the standard treatment for newly diagnosed GBM, although the prognosis remains poor due to the onset of resistance mechanisms that mainly lead to GBM recurrence [4]. A high percentage of GBM patients experience tumor relapse, typically within 6–9 months of initial diagnosis [5]. Moreover, the median progression-free survival time is 5 to 5.6 months for patients with newly diagnosed GBM and 1.7 to 2 months for patients with recurrent disease [6].

The intrinsic heterogenous nature of GBM itself triggers the onset of several mechanisms of resistance to therapy, contributing to tumor recurrence. Among them, the interactions with the tumor microenvironment remain critical.

Cancer-associated fibrosis is increasingly recognized as a central process in the pathophysiology of cancer, particularly in extracranial cancers. Indeed, fibrosis is well established as a mechanism of drug resistance in pancreatic cancer, melanoma, breast cancer, and lung cancer [7,8,9]. In pancreatic cancer, cancer-associated fibroblasts (CAFs) promote collagen, fibronectin, and laminin production by activating pathways as mTOR/4E-BP1 signaling, which contributes to increasing the extracellular matrix (ECM) hardness and density, creating a physical barrier that hinders drug penetration, as well as the migration of activated T cells, impacting the outcomes of T-cell-based immunotherapy in lung cancer [10,11,12]. Moreover, remodeled ECM can restrict the diffusion of oxygen between cells, leading to hypoxia [13]. The establishment of the hypoxic niche adversely influences the tumor sensitivity to chemotherapy while also promoting angiogenesis, invasion, and immunosuppression by activating the transcription of integrin β4 and Akt signaling pathways [14]. Moreover, cancer-associated fibrosis is marked by chronic inflammation within cyto/chemokines (i.e., IL-6, IL-8, IL-17A, and IL-1α), and growth factors (i.e., SDF-1, HGF, PDGF, and TGF-β) are released by both malignant and non-cancer cells, contributing to tumor cell proliferation and invasion. Among growth factors, TGF-β is pivotal in maintaining tissue homeostasis and inhibiting the progression of early tumors into malignant forms by regulating a wide range of cellular processes, including proliferation, differentiation, survival, and adhesion [15]. However, due to their genetic instability, cancer cells possess the ability to evade the suppressive effects of the TGF-β pathway. As a result, TGF-β signaling can paradoxically promote tumor growth, enhance invasion, and support cancer cells in evading the immune system.

Based on this, current therapeutic strategies for several cancers combine the standard-of-care treatment with anti-fibrotic drugs. Notably, a phase I/IIa clinical study (EudraCT Number 2015-003928-31) explored the effect of defactinib (VS-6063), a FAK inhibitor which plays critical roles in integrin-mediated signal transduction, in combination with pembrolizumab (anti-PD-1) therapy in pancreatic cancer, NSCLC, and mesothelioma, assessing the treatment safety, tolerability, and positive clinical activity [16]. Additionally, a phase II study demonstrated that the combination of pirfenidone with nab-paclitaxel and gemcitabine significantly increased the overall survival for patients with metastatic pancreatic cancer [17]. Furthermore, a phase I/II trial highlighted the well-tolerated and promising combination of atezolizumab and pirfenidone for treating patients with non-small cell lung cancer (trial ID: IIT-2020-CAFs) [18].

Nevertheless, it remains unclear whether cancer-associated fibrosis acts as a tumor-promoting or tumor-restraining factor in brain cancer. Indeed, in early-stage tumors, a fibrotic response may slow growth and limit local invasion. Conversely, in more advanced stages, the heightened stiffness and reorganization of the ECM can facilitate tumor growth and proliferation, potentially promoting immune evasion [19,20]. Moreover, it is still not understood how tumor-associated fibrotic alterations in the GBM microenvironment can drive GBM. Both radiotherapy and cytotoxic chemotherapy can initiate the epithelial–mesenchymal transition (EMT) and amplify the signaling of TGF-β. EMT is marked by elevated levels of TGF-β, collagen, fibronectin, α-SMA, and S100A4, suggesting that these molecular changes may foster stromal fibrotic responses in GBM [21,22]. Some researchers have proposed innovative strategies for treating GBM by repurposing existing clinically approved drugs to inhibit EMT. For instance, Kast et al. identified a combination of six drugs (i.e., fenofibrate, quetiapine, lithium, nifedipine, itraconazole, and metformin) that are currently under investigation as potential additive drugs to enhance the effectiveness of chemotherapy in GBM treatment [23]. However, due to the complexity and variability of GBM, additional research is essential to identify which molecular GBM subtypes might benefit the most from these non-antitumor agents. In GBM, molecular studies have identified elevated levels of uPA and uPAR, linking their overexpression to a more invasive tumor phenotype [24]. These studies showed that the urokinase-type plasminogen activator (uPA)—a serine protease that activates a cascade of extracellular proteinases involved in tissue remodeling—together with its receptor uPAR, plays a significant role in the invasion and neovascularization of GBM. Beyond its direct protease activity, uPA also indirectly activates other pro-collagenases that contribute to the breakdown of plasmin-resistant components of the extracellular matrix [25,26]. Further, the upregulation of uPA has been found to inhibit the PI3K–AKT signaling pathway [27]. A peptide corresponding to the region called the connecting peptide of uPA (CPp, residues 135–158) retains the ability to elicit cytoskeletal rearrangements and to direct cell migration [28], while its N-terminal segment (residues 135–143 or 136–143) exhibits remarkable inhibitory properties on cancer cell migration [29,30]. Therefore, we considered uPA-inspired peptides in the present study.

The duality of the role of fibrosis in GBM is influenced not only by the proper nature of the mechanism but also by the significant lack of in vitro pre-clinical models capable of accurately mimicking the GBM–fibrotic milieu found in vivo. Advances in this field are crucial for evaluating therapeutic agents that target the fibrotic tumor microenvironment, as well as for translating preclinical findings into effective therapeutic strategies for GBM patients.

## 2. Results

### 2.1. Development of an in Vitro Model of Glioblastoma-Associated Fibrosis

**Figure 1:**
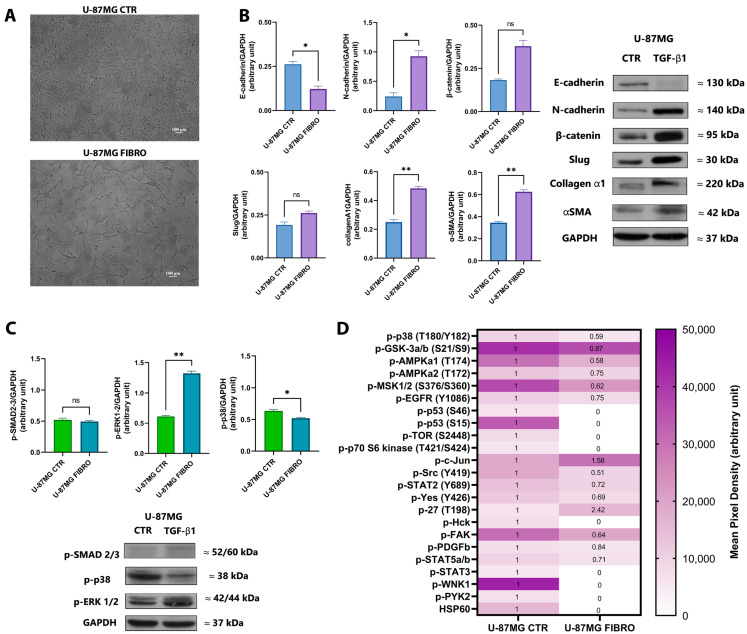
Analysis of fibrotic alterations associated with the in vitro model of fibrosis in GBM. (**A**) Representative bright-field microscopy images of the U-87MG cell line in the absence (on the top) and in the presence (downward) of TGF-β1 (5 ng/mL) after 72 h of treatment. Histograms of the analysis of the expression of the fibrosis-related marker (**B**), the downstream signaling pathways (**C**) in control (CTR), and fibrotic-like (FIBRO) U-87MG cell lines analyzed by Western blotting. Representative immunoblots are also shown. Band densities of the proteins were normalized to the respective loading control GAPDH. Pairwise comparisons were reported (Welch’s *t*-test, * *p* < 0.05, ** *p* < 0.01, ns no significant *p*-value). The heatmap (**D**) represents the panel of the immunodetection of phosphorylated proteins in the control (CTR) and the fibrotic-like (FIBRO) U-87MG cell lines. White boxes correlate with a lower p-kinases concentration and dark-purple boxes with a higher p-kinases concentration (see scale; mean pixel density). The fold-change thresholds within the boxes are reported, calculated as the ratio between the median values of p-kinases from the U-87MG FIBRO cell line, compared to the median values of p-kinases from the U-87MG CTR cell line (posed as CTR at 1). Results are representative of two independent experiments.

To begin our investigation, we established an in vitro cell model to study fibrotic alterations associated with glioblastoma using U-87MG as the commercial GBM cell line. U-87MG cells were exposed to different doses of the well-known pro-fibrotic stimulus TGF-β1 (5 and 10 ng/mL), both in the presence of and without fetal bovine serum (10% and 1%, respectively), over a time course of 24, 48, and 72 h. Our goal was to identify the experimental conditions that most effectively induced a fibrotic-like phenotype in vitro, evaluated by Western blot analysis. Following the interpretation of the obtained results, we focused only on the U-87MG cell line exposed to TGF-β1 at a concentration of 5 ng/mL for 72 h under serum deprivation conditions. Throughout this study, we referred to the untreated U-87MG cell line as *CTR* and the TGF-β1-treated U-87MG cell line as *FIBRO.* Then, we proceeded to investigate the fibrotic-like alterations induction by analyzing the downstream activated pathways. Brightfield microscopy revealed that after 72 h of treatment with TGF-β1 (5 ng/mL), U-87MG cells adopted a fibrotic-like shape, exhibiting a spindle morphology, as shown in Figure 1A. In addition to the phenotypic appearance, we thoroughly investigated the downstream pathways associated with the features of TGF-β1-induced fibrosis, as shown in Figure 1B. Our findings indicated a loss of E-cadherin coupled with the upregulation of N-cadherin and β-catenin, which are functionally linked to the *cadherin switching* observed in fibrosis-related processes. At the transcriptional level, Slug, the transcriptional repressor of E-cadherin, showed overexpression in U-87MG FIBRO, although without reaching statistical significance. Moreover, as typical indicators of fibrosis, we found that collagen type I alpha 1 chain and α-smooth muscle actin expression increased at the protein level. Additionally, in this context, TGF-β1 promoted fibrotic-like alterations via non-SMAD signaling by activating the downstream ERK1/2 pathway in the U-87MG FIBRO cell line (Figure 1C). By performing a multiplex antibody array, we analyzed the relative change in phosphorylated kinase proteins between the U-87MG CTR and FIBRO cell lines. Results are depicted as a heatmap in Figure 1D. Here, we identified a significant differential expression of 22 phosphorylated proteins between the two U-87MG cell lines. Specifically, we confirmed a decrease in p38 phosphorylation, which was previously observed through Western blot analysis, alongside a reduction in mitogen and stress-activated protein kinases 1/2, typically activated by p38. Furthermore, we found that in our in vitro model, TGF-β1 influenced the phosphorylation levels of several kinases associated with different cellular processes, such as (a) epithelial to mesenchymal transition (i.e., the reduction of phosphorylated Ser9/Ser21 in GSK3α/β, which normally stabilizes β-catenin in Wnt signaling; the increase in the activatory Ser63 phosphorylation of c-Jun; and the autophosphorylation of Tyr1068 in EGFR); (b) cell growth (i.e., through the blockade of pro-apoptotic signaling, considering the phosphorylation of Ser15 and Ser46 in p53 led to its destabilization and the reduction of its pro-apoptotic activity, coupled with the dephosphorylation of the inhibitory Ser727 in STAT3, which also contributed to this effect); and (c) migration and invasiveness (i.e., via Src/FAK signaling, which typically allows cell adhesion, hereby unexpectedly lost). These results confirmed the almost complete accuracy of our model.

### 2.2. Fibrotic-like U-87MG Cells Exhibit Increased Resistance to TMZ

**Figure 2:**
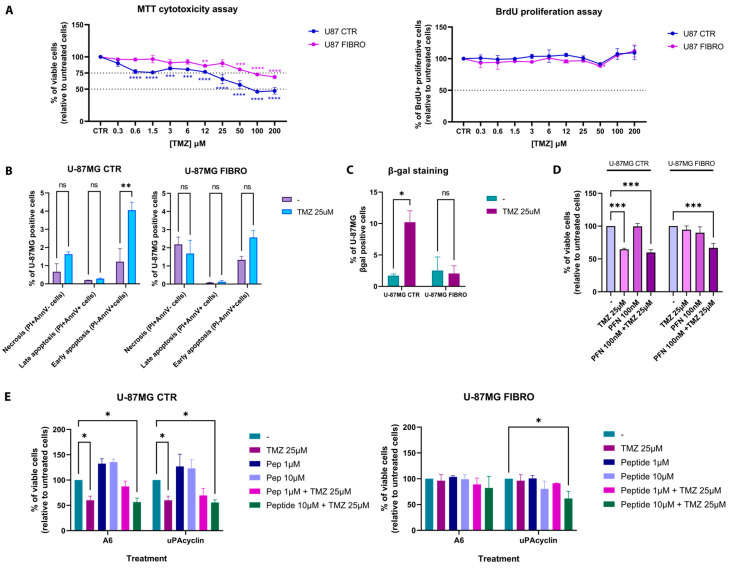
Effects of TMZ and anti-fibrotic compounds in an in vitro model of fibrosis in GBM. (**A**) CTR and FIBRO U-87MG cell lines were treated with different concentrations of TMZ (from 0 to 200μM) for 72 h. On the left, the graph shows the effect of TMZ on cell viability, analyzed by the MTT assay. On the left, the graph shows the effect of TMZ on cell proliferation, analyzed by the BrdU ELISA assay. (**B**) CTR and FIBRO U-87MG cell lines were treated with TMZ (25 μM) for 72 h. The bar graph shows the effect of TMZ on necrosis and late and early apoptosis, analyzed by Annexin V–PI staining by flow cytometry. In (**C**), the effect of TMZ on senescence was analyzed by SA-β-gal staining by flow cytometry. (**D**) CTR and FIBRO U-87MG cell lines were pre-treated with PFN (100 nM) overnight and then treated or untreated with TMZ (25µM). The histogram graph shows the percentage of viable cells analyzed by MTT assay. (**E**) CTR and FIBRO U-87MG cell lines were treated with A6 and uPAcyclin at different concentrations (1 and 10µM) in the presence or absence of TMZ (25µM). The histogram graph shows the percentage of viable cells analyzed by MTT assay. Pairwise comparisons were reported (one-way or two-way ANOVA, * *p* < 0.05, ** *p* < 0.01, *** *p* < 0.001, **** *p* < 0.0001, ns: not significant). Results were representative of three independent experiments, expressed as mean ± SD.

Once we detected the experimental conditions and the associated pathways involved in the distinct phenotypes (CTR vs. FIBRO), we proceeded to analyze the response of the U-87MG FIBRO cell line to temozolomide (TMZ) treatment in a dose–response manner (0–200 µM) after 72 h to ascertain our hypothesis regarding the resistance of fibrotic-like GBM cells to TMZ exposure. As expected, the results from the MTT cytotoxicity assay revealed a significant resistance of the fibrotic-like U-87MG cells to TMZ when compared to U-87MG CTR (Figure 2A). Indeed, the control cell line demonstrated a higher sensitivity to TMZ, exhibiting a response even at low concentrations of the drug (predicted EC_50_ = 35 µM). In contrast, the fibrotic-like cell line showed a statistically significant reduction in cell viability only at higher concentrations of TMZ, yet the EC_50_ was never achieved (predicted EC_50_ = 351 µM). In terms of the proliferative rate, TMZ did not affect the proliferation in either of the cell lines (Figure 2A). Following a preliminary investigation of TMZ dose–response using an MTT assay, we decided to focus on a specific concentration of TMZ (25 µM), aiming to confirm the lower impact of TMZ on the U-87MG FIBRO cell line by examining the two primary responses elicited by TMZ-induced DNA damage in GBM cells: apoptosis and senescence. We selected the 25 µM concentration, since, even though it does not reach the IC_50_ in the U-87MG CTR, the fibrotic-like cells still remain viable. Through FACS analysis, we demonstrated that TMZ at a concentration of 25 µM induced early apoptosis and senescence (two key processes of the TMZ mechanism of action) in control U-87MG cells. By contrast, fibrotic-like U-87MG cells did not exhibit a response to these TMZ-induced mechanisms (Figure 2B,C). These results confirmed the development of TMZ resistance in our model of fibrotic-like GBM cells. At this point, we wondered whether the pre-treatment with the anti-fibrotic drug could enhance the responsiveness of fibrotic-like cells to TMZ. To restore sensitivity to TMZ, we tested pirfenidone (PFN), an anti-fibrotic agent known for inhibiting the TGF-β1 pathway and recently applied in combination with various therapeutic strategies for non-small cell lung cancer, pancreatic cancer, and colorectal cancer [31,32,33,34]. Our findings indicated that overnight pre-treatment with PFN (100 nM) coupled with TMZ treatment (25 µM) can induce a comparable level of cell death in fibrotic-like U-87MG cells, nearly matching the effects of TMZ alone (25 µM) in the control cell line (CTR vs. FIBRO: 64% vs. 67% of cell viability) (Figure 2D). This result was particularly noteworthy, considering PFN by itself does not exhibit cytotoxicity in the U-87 CTR cell line, nor does it produce any non-specific effects. Indeed, PFN did not enhance the sensitization of U-87MG CTR cells to TMZ, where tumor-associated fibrotic changes were absent. According to the data obtained, we explored the potential additive or synergistic effects of novel anti-fibrotic peptides in combination with TMZ treatment.

To this aim, we evaluated two different urokinase-derived peptides: (i) the octapeptide A6 (Ac-KPSSPPEE-NH_2_), corresponding to uPA residues 136–143, endowed with clearcut anti-angiogenetic and anti-metastatic activities in mice [35], and (ii) uPAcyclin (Ac-KP[ESPPEELK]-NH_2_), a recently developed cyclic decapeptide derived from A6. uPAcyclin design, conformational analysis, and binding assay were carried out as outlined in the study by Belli et al. [36]. Moreover, it has been evaluated in both in vitro and in vivo models of lung cancer metastasis and fibrosarcoma. This novel urokinase-derived decapeptide was able to interfere with the ability of tumor cells to migrate and invade by altering the phenotype of the cancer-associated fibroblasts (CAFs). Indeed, CAFs play a pivotal role in facilitating cancer cell invasion within the stromal extracellular matrix through integrin-linked mechanisms and alphaV–integrin-dependent matrix reorganization. Interestingly, uPAcyclin was also able to inhibit the vasculogenic mimicry of glioblastoma cells [37].

The two peptides were evaluated in our in vitro model in time-course (24 h, 48 h, and 72 h) and dose–response (10 µM, 1 µM, and 100 nM) manners, in the presence or absence of TMZ (25 µM). We chose this window of concentrations because previous studies showed that the biological effects of A6 typically occur within the micromolar range, and no cytotoxicity was observed at concentrations up to 100 µM in vitro [38]. Here, we presented the most promising concentrations of the two peptides (10 µM and 1 µM) after 72 h of treatment. The results from the cytotoxicity assay in Figure 2E indicated that the peptides did not induce cell death in U-87MG CTR cells (left panel), where any effects on cell viability were solely due to the TMZ treatment, especially given the absence of established fibrosis-related mechanisms. Indeed, the % of cell viability in this condition (55.75% ± 5.7%) overlapped with those achieved by TMZ treatment alone (56.33% ± 5.7%). On the contrary, we documented a significantly decreasing trend in cell viability for fibrotic-like U-87MG cells treated when TMZ was supplemented with uPA cyclin (10 µM), compared to treatments with TMZ alone (25 µM).

## 3. Discussion

The current findings underscore the significant role of tumor-associated fibrotic-like alterations in contributing to drug resistance in GBM. Using a two-dimensional in vitro model induced by TGF-β1, we explored the activation of fibrotic pathways and their influence on resistance to TMZ. Specifically, we observed that the TGF-β1-induced fibrotic phenotype correlated with E/N-cadherin switching, increased expression of collagen and α-SMA, and the activation of SMAD-independent signaling pathways (Figure 1B,C). Additionally, TGF-β1 was found to activate or inhibit several kinases linked to migration, apoptosis, and cell growth. If some results were in line with established findings on TGF-β1-mediated fibrosis in cancer, elsewhere, the pattern was counterintuitive at first glance (Figure 1D). In detail, p38, and consequently the MSK1/2 complex, is well-known to be implicated in a plethora of cellular functions, and according to acute or persistent stimuli, p38 regulates different mechanisms [39,40]. In our experimental setting, TGF-β1 led to the deactivation of p-p38 and the relative cascade, which was very likely considering U-87MG cells were exposed to a low dose of TGF-β1 (i.e., 5 ng/mL) for a prolonged time (i.e., 72 h)—two conditions reflecting non-acute treatment. Inflammatory cytokines such as TNF, IL-6, and IL-1β are prototypic activators of p38α, mainly when administered at a high concentration for a short time [40]. Moreover, here, we highlighted that TGF-β1 induces the activation of SMAD-independent signaling pathways. According to that, it has been reported that TGF-β1 prevents pro-inflammatory cytokine production through the inhibition of p38 mitogen-activated protein kinase (MAPK) and NF-kB. Other investigations indicated that TGF-β1, as well as in our setting, activates ERK, which, in turn, can up-regulate MAPK phosphatase-1, thereby inactivating p38 MAPK [41]. This could explain the deactivation of the p38 signaling in our setting. Likewise, in our in vitro model of glioblastoma-associated fibrosis, TGF-β1 unexpectedly reduced FAK activation, probably due to the late analysis time (i.e., 72 h of TGF-β treatment). Indeed, FAK phosphorylation levels fluctuated over time, and this oscillation was particularly evident in the context of cell migration and focal adhesion dynamics. While our result is inconsistent with the well-recognized role of FAK in cell adhesion and migration, FAK has been reported to be transiently activated during TGF-β treatment but becomes dephosphorylated during prolonged exposure, leading to changes in cytoskeletal dynamics [42]. In line with our model, in glioma cells, Wendt et al. found that TGF-β drives mesenchymal traits via FAK/PI3K, but following chronic exposure to TGF-β, FAK may undergo de-phosphorylation or be internalized/degraded, with the loss of anchor signal and the suppression of downstream factors [43]. In light of that, the observed alterations induced by TGF-β1 in the U-87MG FIBRO cell line led to a significant increase in TMZ resistance, affecting cytotoxicity, senescence, and apoptosis, aligning with previous observations that fibrotic-like features can enhance tumor cell survival (Figure 2A–C) [44,45,46]. As an added value, our results also indicated that drugs currently in clinical practice, such as pirfenidone, can effectively target the fibrotic phenotype of GBM cells, thereby increasing their sensitivity to TMZ (Figure 2D). Furthermore, novel anti-fibrotic drugs were tested. Among them, uPAcyclin appeared to be the most promising one. Preclinical studies have shown that A6 has anti-angiogenic, anti-migratory, anti-invasive, and anti-metastatic effects, even in vivo when combined with cisplatin in GBM models [31,35,47]. A phase II study revealed that administering A6 at a daily dose of 300 mg for 28 days is significantly associated with improved progression-free survival in patients diagnosed with ovarian, peritoneal, and fallopian tube carcinoma [38]. A6 works by binding to and activating signaling pathways associated with CD44, a well-known membrane protein involved in cell–cell and cell–matrix adhesion [48]. As reported, A6 enhances the activity of focal adhesion kinase (FAK), demonstrated by its phosphorylation at tyrosine 397, which initiates the cytoskeleton reorganization. Furthermore, the peptide A6 binds to a specific site within the cytoplasmic domani of CD44 with high affinity, triggering the activation of Src [49]. This interaction leads to the Ras-dependent activation of the MAP kinase cascade, which involves the phosphorylation of MEK and STAT5 [50]. In addition, A6 analogs, among which is uPAcyclin, have been demonstrated to interact and exert their action through the inhibition of the αv integrin [36]. Mechanistically, this aligns with our fibrotic model, in which TGF-β1 deactivates the FAK–Src–MEK axis (Figure 1D), probably leading to the disruption of cell adhesion and subsequently increasing cell migration and invasiveness. In this context, our findings revealed for the first time that the peptide uPAcyclin is able to restore fibrotic-like glioblastoma cell sensitivity to TMZ (Figure 2E). Interestingly, uPAcyclin exhibits greater cytotoxicity activity when used in combination with TMZ, compared to the clinical candidate peptide A6. These preliminary in vitro findings suggest that uPAcyclin could serve as the lead compound in the development of promising therapeutic strategies against GBM-related fibrosis-like mechanisms.

However, we only focused on the cytotoxic potential of the compound, and we did not explore its effect on fibrosis-related pathways. Therefore, further functional studies are required to mechanistically confirm its role in overcoming drug resistance. Nevertheless, the main aim of this study was to establish a straightforward, readily reproducible, and efficient in vitro GBM-associated fibrotic alterations model. Thus, these preliminary results support the reliability of our model. However, other limitations are worth considering. While the 2D in vitro model facilitated a detailed molecular characterization of the pathways related to fibrosis, it did not accurately replicate the complex three-dimensional architecture and mechanical properties of the GBM microenvironment. To address this limitation, further studies should employ advanced 3D culture models that consider the interactions between GBM cells and stromal components. This will help to more closely mimic how the tumor microenvironment, such as the release of the TGF-β itself and other fibrotic stimuli, induces fibrosis in GBM cells. Very recently, Amofa et al. refined a 3D model of TGF-β-driven invasion in tumors derived from GBM stem cells (GSCs) through the application of 3D hyaluronic acid matrices [51]. Since we have yet to explore the functional effects of uPAcyclin decapeptide in our experimental setup, this model could be particularly relevant, especially since U-87MG cells are well-suited for 3D models as well. Additionally, in vivo studies will be crucial for validating the translational potential of the identified anti-fibrotic compound uPAcyclin and its putative synergy with TMZ, aligning with recent findings from Watson et al. [52] (Supplementary Figure S1).

In conclusion, this work presents a promising strategy for sensitizing resistant GBM and improving therapeutic outcomes by introducing a novel in vitro model of fibrotic-like alterations in GBM for the development of combinatory therapeutic strategies. Taken together, these findings contribute to our understanding of the field and provide a rationale for designing new generations of anti-fibrotic and anti-cancer drugs targeting GBM. These advances can pave the way for achieving a broader therapeutic window, reducing off-target effects associated with fibrosis, and ultimately enhancing patient prognosis.

## 4. Materials and Methods

### 4.1. In Vitro Model

The U-87MG cells were cultured in EMEM (Gibco^®^, Thermo Fisher Scientific, Waltham, MA, USA) supplemented with 10% or 1% heat-inactivated FBS (Gibco^®^, Thermo Fisher Scientific, Waltham, MA, USA), 1% L-glutamine (Aurogene, Rome, Italy), 1% antibiotic mixture (Aurogene, Rome, Italy), 1% sodium pyruvate (Aurogene, Rome, Italy), and 1% non-essential amino acids (MEM NEAA, Gibco^®^, Thermo Fisher Scientific, Waltham, MA, USA) and maintained at 37 °C in a humidified 5% CO_2_ atmosphere. For the in vitro model, the U-87MG cells were seeded at low density (10.000 cells/cm^2^) in standard 6-well plates (Corning^®^, New York, NY, USA, #3370) in EMEM-complete 10% FBS for 24 h, then the medium was changed with EMEM-complete 1% FBS, and then treated without or with recombinant human TGF-β1 (5 ng/mL; BioLegend, San Diego, CA, USA #781802) in a time-course manner (until 72 h) to respectively generate the control cell line (i.e., U-87MG CTR) and the fibrotic cell line (i.e., U-87MG FIBRO). Afterwards, cells were trypsinized (Trypsin-EDTA 0.25%, #25200056, Gibco^TM^, Thermo Fisher Scientific, Waltham, MA, USA) and re-plated at low density (10.000 cells/cm^2^) in 96-well flat-bottom plates (Corning^®^, New York, NY, USA; #3736) with fresh medium for additional days (in a time-course manner until 72 h) in the absence or presence of different drugs: temozolomide (Sigma–Aldrich, Darmstadt, Germany, T2577-10) in a dose–response of 0–200 µM; pirfenidone (PFN; 100 nM; Sigma–Aldrich, Darmstadt, Germany, P2116); and uPA peptides (A6, uPAcyclin; 10 µM and 1 µM) [34].

### 4.2. Peptide Synthesis

Peptides A6 (Ac-KPSSPPEE-NH_2_) and uPAcyclin (Ac-KP[ESPPEELK]-NH_2_) were synthesized by ultrasound-assisted solid-phase peptide synthesis (US-SPPS) on Rink amide resin (0.1 mmol scale), using the Fmoc/*t*Bu protection strategy, as previously described [53]. Fmoc deprotection was performed with 20% piperidine in DMF, and amino acid couplings were carried out with N^α^-Fmoc amino acids, HBTU/HOBt (3 equiv), and DIEA (6 equiv) under ultrasonic agitation. For uPAcyclin, an additional on-resin cyclization step was performed using PyAOP (3 equiv) and DIEA (6 equiv) for 12 h rt [54]. Peptides were cleaved and fully deprotected using a TFA/TIS/H_2_O mixture (95:2.5:2.5, *v*/*v*/*v*), then purified by preparative RP-HPLC on a C_18_ column using a linear ACN gradient (10–90%, 0.1% TFA) over 20 min, with a flow rate of 10 mL/min. After lyophilization, purity (>95%) was confirmed by analytical UHPLC, and peptide identity was verified by ESI-MS in positive ion mode (*m/z* 200–2000).

### 4.3. Western Blotting

The U-87MG cells were washed with PBS (Gibco^®^, Thermo Fisher Scientific, Waltham, MA, USA), harvested, and lysed in ice-cold RIPA lysis buffer (50 mM Tris-HCl; 150 mM NaCl; 0.5% Triton X-100; 0.5% deoxycholic acid; 10 mg/mL leupeptin; 2 mM phenylmethylsulphonyl fluoride; and 10 mg/mL aprotinin, phosphatase inhibitor, and protease inhibitor). After centrifugation (13,000 rpm for 20 min at 4 °C) to remove cell debris, proteins were quantified. Approximately 25 µg of proteins were separated on 10% SDS-PAGE at 90 V for 1 h and at 120 V for 1 h and then transferred to a nitrocellulose membrane. After blocking with 5% nonfat dried milk powder (PanReac AppliChem, Darmstadt, Germany) in Tris-buffered saline containing 0.05% Tween-20 (TBST) for 1 h at room temperature, the membranes were incubated overnight with the following primary antibody Abs: Ab anti-E-cadherin (Cell signaling, CS3195T; Danvers, MA, USA), Ab anti-N-cadherin (Cell signaling, CS13116; Danvers, MA, USA), Ab anti-β-catenin (Cell signaling, CS9562; Danvers, MA, USA), Ab anti-Slug (Cell signaling, CS9585T; Danvers, MA, USA), Ab anti-COL1A1 (Cell signaling, CS72026; Danvers, MA, USA), Ab anti-αSMA (Cell signaling, CS19245; Danvers, MA, USA), Ab anti-p-ERK1/2 (Cell signaling, CS9101; Danvers, MA, USA), Ab anti-p-p38 (Cell signaling, CS4511; Danvers, MA, USA), Ab anti-p-SMAD2/3 (Cell signaling, CS8828T; Danvers, MA, USA), and Ab anti-GAPDH (Santa Cruz, SC32233; Dallas, TX, USA). Immunodetection of specific proteins was carried out with horseradish peroxidase-conjugated donkey anti-mouse IgG (Bio-Rad, Hercules, CA, USA), using the enhanced chemiluminescence (ECL) system (Thermo Fisher Scientific, Waltham, MA, USA) according to the manufacturer’s instructions, and then exposed to X-ray films (Thermo Fisher Scientific, Waltham, MA, USA). Western blot data were analyzed using Photoshop software to determine the optical density (OD) of the bands. The OD readings of proteins were expressed as a ratio relative to GAPDH. Bar graphs and statistical analysis were generated using GraphPad Prism 8.0 software.

### 4.4. Human Phospho-Kinase Array

The Proteome Profiler^TM^ Array Human Phospho-Kinase Array Kit (R&D systems, ARY003B; Minneapolis, MN, USA) was applied for the parallel determination of the relative levels of protein phosphorylation. A total of 320 µg of cell lysates of the control and fibrotic U-87MG cell lines was added to the membranes overnight at 4°C on a rocking platform shaker. After several washes, the detection antibody cocktails were added and then washed. Immunodetection of phosphorylated proteins was carried out with the provided Chemi Reagent Mix and then exposed to X-ray films (Thermo Fisher Scientific, Waltham, MA, USA). Data were analyzed using Photoshop software to determine the optical density (OD) of the dots. The OD readings of phosphorylated proteins were expressed as a ratio relative to the control. Heatmap and statistical analysis were generated using GraphPad Prism 8.0 software.

### 4.5. Cytotoxicity Assay

The MTT (3-(4,5-dimethylthiazol-2-yl)-2,5-diphenyl tetrazolium bromide (Sigma–Aldrich, Darmstadt, Germany; #M5655) assay was performed on U87-MG cells after specific treatment to evaluate cytotoxicity. U-87MG cells were seeded in 96-well plates and treated with various concentrations of the compound, as mentioned above, in triplicate. After 72 h of treatment, 15 µL of serum-free medium containing 0.5 mg/mL MTT (Sigma–Aldrich, Darmstadt, Germany; #M5655) was added to 150 µL of cell suspension in each well and incubated for 4 h in a humidified incubator. The water-insoluble formazan was dissolved by adding 150 µL of lysis buffer solution (HCl 0,1M, Triton X-100 10%, isopropanol) for 18 h, and the absorbance was detected at a wavelength of 570 nm using the microplate reader Infinite^®^ M200 PRO Microplate Reader (Tecan Trading AG, Männedorf, Switzerland). Dose–response curves, bar graphs, and statistical analysis were generated using GraphPad Prism 8.0 software.

### 4.6. Flow Cytometry

#### 4.6.1. Apoptosis Assay

The Annexin V FITC Apoptosis Detection Kit (Cat. AD10, Dojindo Laboratories, Rockville, MD, USA) was employed to evaluate U87-MG cell apoptosis after treatment with temozolomide. After detaching cells, the cell pellet was resuspended in Annexin V buffer 1x. A total of 5 µL of Annexin V FITC was added to the cell suspension and incubated for 10 min at room temperature with light protection. After centrifugation and washing, the cell pellet was resuspended in Annexin V buffer and stained with 5 µL of propidium iodide PE solution without washing before FACS analysis using a FACS VERSE flow cytometer (BD Biosciences, Franklin Lakes, NJ, USA). Bar graphs and statistical analysis were generated using GraphPad Prism 8.0 software.

#### 4.6.2. Senescence Assay

The U-87MG cells (treated as mentioned above) were also stained for SPiDER-βGal (Cat. SG02, Dojindo Laboratories, Rockville, MD, USA). A total of 200 μL of 1 μmol/L SPiDER-βGal working solution in HBSS (Gibco^®^, Thermo Fisher Scientific, Waltham, MA, #14175095) was added to the cell pellet and incubated for 15 min at 37 °C in the dark in a humidified incubator without washing before FACS analysis. For each test, cells were analyzed using a FACSVerse flow cytometer (BD Biosciences, Swindon, UK). Bar graphs and statistical analysis were generated using GraphPad Prism 8.0 software.

#### 4.6.3. Proliferation Assay

A colorimetric immunoassay, based on the measurement of BrdU incorporation during DNA synthesis, was performed for the quantification of cell proliferation (Cell Proliferation ELISA, BrdU colorimetric, 11647229001, Roche; Basel, Switzerland). A 20 μL/well BrdU labeling solution was added to U-87MG cells (treated as mentioned above) overnight at 37 °C in the dark in a humidified incubator. After the removal of the labeling medium, cells were fixed and denatured. After the washes, 100 μL/well of anti-BrdU-POD working solution was added. For the detection, 100 μL/well substrate solution was added up to 30 min. The absorbance was detected at a wavelength of 370 nm using the microplate reader Infinite^®^ M200 PRO Microplate Reader (Tecan Trading AG, Männedorf, Switzerland). Dose–response curves and statistical analysis were generated using GraphPad Prism 8.0 software.

### 4.7. Statistical Analysis

In all experiments shown, statistical analysis was performed using the GraphPad Prism 8.0 software for Windows (GraphPad software). For each type of assay or phenotypic analysis, data obtained from multiple experiments were calculated as mean ± SD and analyzed for statistical significance using appropriate tests. For the *t*-test (Welch’s *t*-test) or analysis of variance (one-way or two-way ANOVA) for multiple comparisons, *p*-values <0.05 were considered significant (* *p* < 0.005, ** *p* < 0.01, *** *p* < 0 0001, and **** *p* < 0.0001).

## Data Availability

The original contributions presented in this study are included in the article. Further inquiries can be directed at the corresponding authors.

## Supplementary Materials

The following supporting information can be downloaded at: https://www.mdpi.com/article/10.3390/ijms26136121/s1.

## Abbreviations

The following abbreviations are used in this manuscript:

CAF	Cancer-associated fibroblast
CNS	Central nervous system
CTR	Control
DIEA	N,N-diisopropylethylamine
ECM	Extracellular matrix
EMT	Epithelial-to-mesenchymal transition
ESI-MS	Electrospray ionization mass spectrometry
FACS	Fluorescence-activated cell sorter
FAK	Focal adhesion kinase
FIBRO	Fibrotic
GBM	Glioblastoma
HGF	Hepatocyte growth factor
MEK	Mitogen-activated protein kinase kinase
MTT	3-(4,5-dimethylthiazol-2-yl)-2,5-diphenyltetrazolium bromid
NSCLC	Non-small cell lung cancer
PDGF	Platelet-derived growth factor
PyAOP	(7-Azabenzotriazol-1-yloxy) tripyrrolidinophosphonium hexafluorophosphate
PFN	Pirfenidone
RT	Radiation therapy
SDF-1	Stromal cell-derived factor-1
STAT3	Signal transducer and activator of transcription 3
TFA	Trifluoroacetic acid
TGF-β	Transforming growth factor beta
TIS	Triisopropylsilane
TMZ	Temozolomide
UHPLC	Ultra-high-performance liquid chromatography
uPA	Urokinase-type plasminogen activator
uPAR	Urokinase-type plasminogen activator receptor
