# Supplementary material for: Establishment of U-87MG Cellular Fibrosis as a Novel in Vitro Model to Analyze Glioblastoma Cells’ Sensitivity to Temozolomide
Source: Int J Mol Sci. 2025 Jun 25;26(13):6121. doi: 10.3390/ijms26136121 (PMC12249993; doi:10.3390/ijms26136121)
Supplement: Supplementary file 1 [file ijms-26-06121-s001.zip › ijms-3671383-supplementary.pdf]

Supplementary Figure S1.

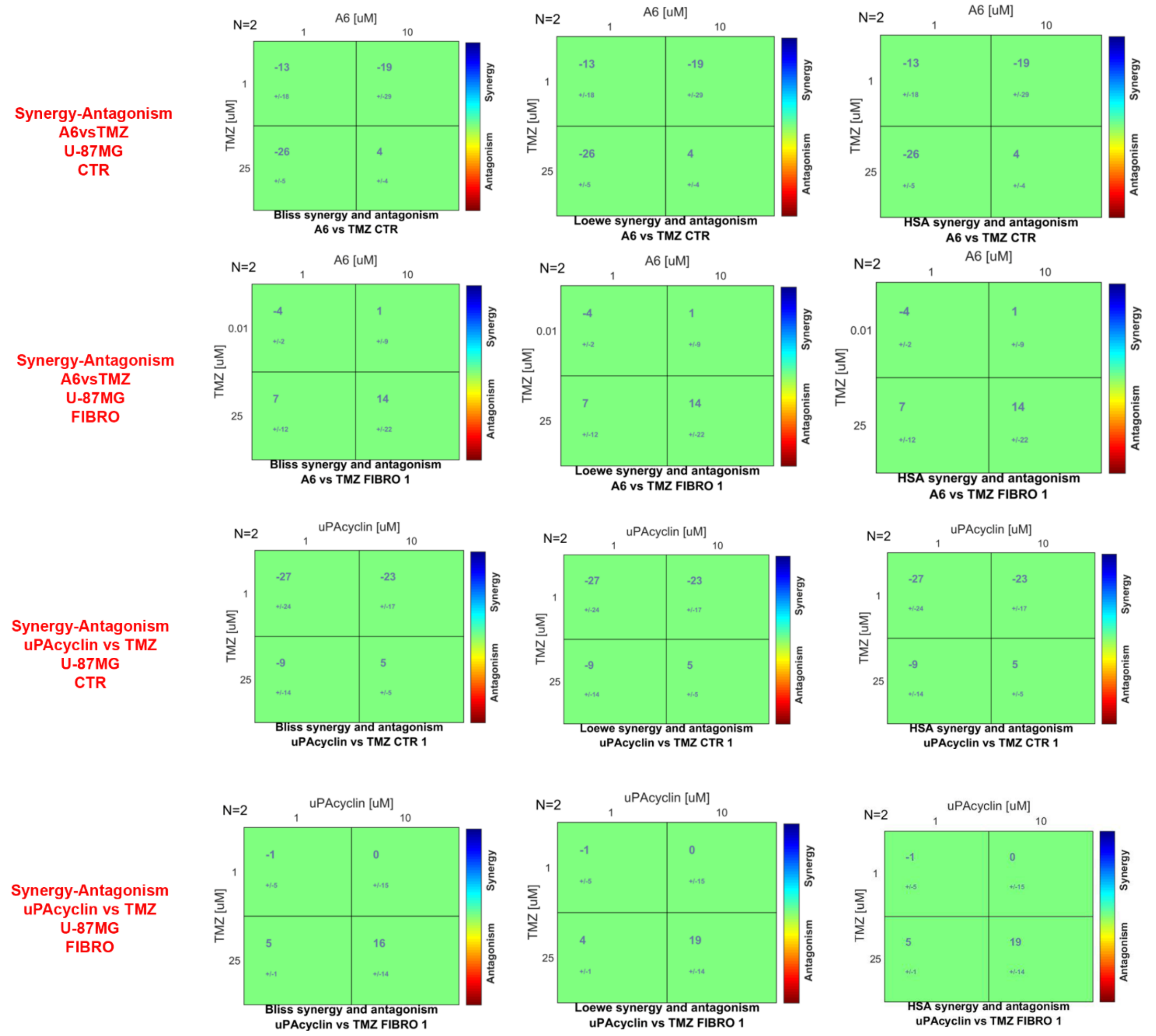


*Figura S1. The synergy distribution matrix obtained by applying Combenefit software in U-87MG CTR and FIBRO cell lines between A6 or uPAcyclin (1uM, 10uM) and TMZ (25uM). The phenotypic response is expressed chromatically, where synergistic interaction is represented by blue, antagonistic interaction is represented by red, no interaction in green. Data were obtained from two independent experiments (n = 2). Three synergy models (Bliss, Loewe, HSA) have been performed .*
